# Effect of Whole-Body Vibration Training on Hemorheological Blood Indices in Young, Healthy Women

**DOI:** 10.3390/ijerph20043232

**Published:** 2023-02-12

**Authors:** Halina Gattner, Justyna Adamiak, Anna Piotrowska, Olga Czerwińska-Ledwig, Sylwia Mętel, Magdalena Kępińska-Szyszkowska, Wanda Pilch

**Affiliations:** 1 Faculty of Physiotherapy, University of Physical Education in Krakow, Jana Pawła II Avenue 78, 31-571 Krakow, Poland; 2 Institute of Applied Sciences, Faculty of Physiotherapy, University of Physical Education in Krakow, Jana Pawła II Avenue 78, 31-571 Krakow, Poland; 3 Department of Chemistry and Biochemistry, Faculty of Physiotherapy, University of Physical Education in Krakow, Jana Pawła II Avenue 78, 31-571 Krakow, Poland

**Keywords:** whole-body vibration training (WBVT), hemorheology, elongation index, aggregation index, blood plasma volume, healthy women

## Abstract

The aim of the study is to assess the effect of single and 12-week WBVT and training without vibration on changes in hemorheological blood indices and plasma fibrinogen levels in young, healthy women. Three groups are distinguished: the experimental group—participating in WBVT (*n* = 17); the comparison group—implementing the same physical exercise protocol without the vibration factor (*n* = 12); and the control group—no intervention (*n* = 17). In the experimental and comparison group, blood is collected before and after the first and last training, while in the control group, blood is collected twice, 3 months apart. After a series of WBVT, a significant decrease in the mean erythrocyte volume and mean hemoglobin mass in erythrocytes, as well as a slight increase in the mean erythrocyte hemoglobin concentration, is found, and the effect of the last training is a significant decrease in plasma volume. Under the influence of repeated WBVT, there is an increase in erythrocyte deformability at low shear stress and an increase in the aggregation amplitude. The study shows that WBVT improves blood flow in the vessels and does not affect erythrocyte aggregation and the level of fibrinogen, which confirms the safety of this form of exercise.

## 1. Introduction

Whole-body vibration training (WBVT) is based on neuromuscular stimulation of the body by mechanical vibrations in combination with performing static or dynamic exercises on a vibration platform [1]. Increased activation of skeletal muscles is associated with the development of the tonic vibration reflex, which causes cyclical and short-term changes in the length of the tendon-muscle complex [2,3]. Vibrations with appropriate parameters increase the effect of gravitational forces because of high accelerations transferred cyclically to the body, which results in specific training reactions, often compared to strength exercises [1,4]. Since WBVT takes less time than traditional training, it can also be a valuable supplement [1]. The main effects observed after using WBVT include, above all, an increase in muscle strength [5], improvement in bone density [6], reduction in adipose tissue [7], and stimulation of blood circulation [8,9] and hormonal balance [10,11]. However, the mechanical action of vibrations in combination with physical exercises has not been thoroughly explained so far, especially in the context of internal changes in the body under the influence of WBVT.

Physical activity is one of the factors that may affect blood hemorheology [12,13]. Under the influence of a single, long-term physical effort, plasma volume decreases by up to 15% (hemoconcentration: increase in hematocrit and blood viscosity, while the total number and volume of erythrocytes do not change substantially) [13]. A frequently observed effect of intensive physical exercise is a decrease in the erythrocytes’ deformability [14,15,16] and an increase in their ability to form blood cell aggregates, which in turn increases blood flow resistance, especially in small blood vessels [17,18,19]. The body’s response to regular physical training is an increase in plasma volume, even by 20%, which is associated with a decrease in hematocrit and blood viscosity. Long-term effects of physical exercise also include increased deformability and reduced ability of erythrocytes to aggregate, which facilitates blood flow [20,21,22].

The mechanical vibrations generated by vibrating platforms related to blood flow and its rheological properties, such as erythrocyte deformability and aggregation capacity, have not been described so far. There is also little information on the impact of WBVT on hematological blood indices. Therefore, the aim of the study was to assess the comparative effect of single and 12-week whole-body vibration training and training without the vibration factor on changes in the level of hemorheological blood indices and plasma fibrinogen concentration.

## 2. Materials and Methods

### 2.1. Participants

Fifty-four healthy, non-training women aged 19–25 (mean age 20.48 ± 1.72) who met the following inclusion and exclusion criteria were qualified to participate in the study (Table 1).

The study was conducted between January 2017 and October 2018 in accordance with the Declaration of Helsinki and approved by the Bioethics Committee at the District Medical Chamber in Krakow (consent number 224/KBL/OIL/2016). The project has been registered in the clinical trials database (trial ID: ACTRN 12621000114842). The subjects were instructed not to change their diet and not to undertake additional physical activity during the project. The women were also informed about the purpose and plan of the research, potential side effects, and the possibility of resigning from participation in the research project at any time.

**Figure 1:**
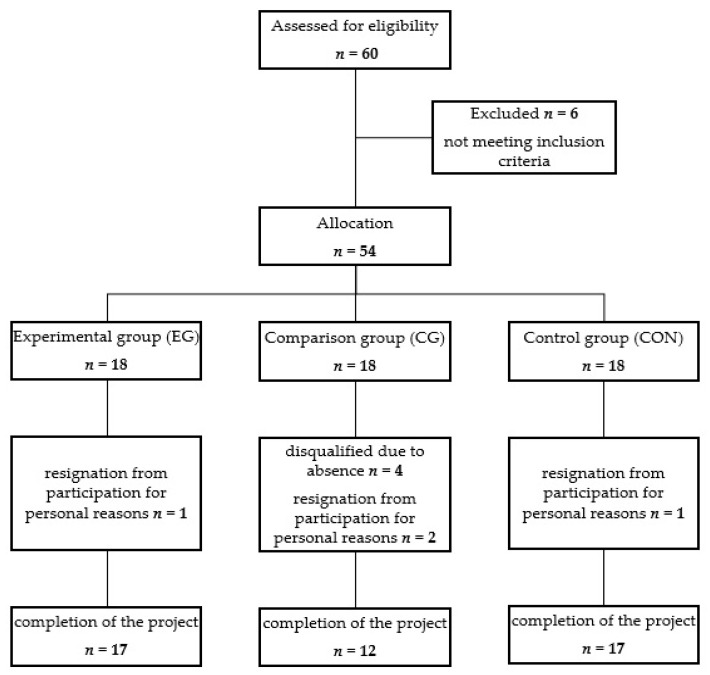
Study flow diagram.

Allocation of subjects into different groups was done through volunteer sampling—the participants were recruited via posted advertisement at the university and assigned to the group to which they volunteered. Three groups were selected from among the people registered and qualified for the study: the examined group participating in the whole-body vibration training (experimental group, EG), the comparative group implementing the same training scheme in the form of group classes without the vibration factor (comparison group, CG), and the control group not subjected to any physical activity (control group, CON). The scheme of the recruitment process is shown in Figure 1.

The characteristics of the selected groups are presented in Table 2.

### 2.2. Study Protocol

The protocol included two series of studies: preliminary and main. The initial examination in each group consisted of an interview and medical examination, body composition and nutrition analyses, and assessment of the level of physical activity. The main study in the experimental and comparison group included 12-week training with four blood samples for analysis: I—before one training, II—after one training, III—before the last training, and IV—after the last training. The preliminary study was conducted within a month before the main study. In the control group, blood sampling was carried out twice with an interval of 3 months. The research project was carried out in two stages, in two consecutive years (stage I—the experimental group performing exercises on a vibration platform and the control group-not subjected to any intervention; stage II—the comparison group performing the same physical exercise regimen, without the vibration factor). Recruitment and selection of people for each group from among the volunteers registered for the study took place separately.

### 2.3. Exercises Program

**Figure 2:**
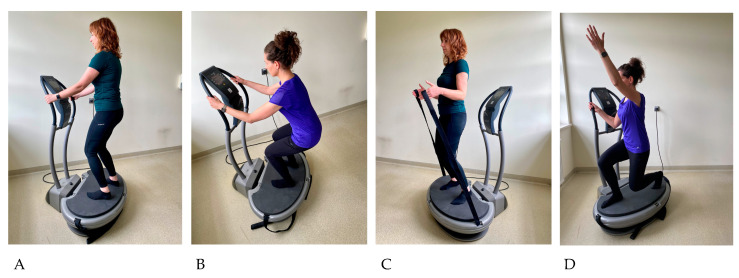
Exercises performed in the main part of the training session on a vibrating platform. (**A**) Front standing position on the platform with a two-handed safety grip on the platform railings (exercise no. 1); (**B**) Dynamic squat (exercise no. 2) and static squat (exercise no. 6) to the angle of 90° in the knee joints; (**C**) Back standing position on the platform while holding the tapes in the hands (exercise no. 3) in the closed biokinetic chain for the upper and lower extremities’; (**D**) Dynamic squat in a lunge with a one-handed safety grip and raising the opposite upper limb—exercise performed twice, once with the right and once with the left lower limb (exercise no. 4 and 5).

The training program in both groups included 36 sessions of physical exercise performed 3 times a week for 12 weeks. In EG, each session was conducted individually and included a short warm-up, a main part carried out on a vibration platform, and a final part consisting of relaxation exercises. Women from CG performed the same exercise regimen in the form of supervised classes in groups of 3–4 people (the first and last training was carried out individually), without the use of a vibrating platform in the main part. The warm-up consisted of 3 exercises carried out in a high position, 6 repetitions each: transition from a half-squat to standing position with simultaneous raising of the arms sideways, alternating lunges forward with the right and left lower limbs while raising the arms sideways to the angle of 90 degrees, and dorsiflexion of the hands, hip rotation to the right and left. In the main part, the subjects performed 3 static and 3 dynamic exercises, each lasting 1 min in a high position on a vibration platform [Figure 2]. Between each exercise there was a 1 min rest interval during which the women walked around the room. During dynamic squats, the pace was set using a metronome (25 movements per minute). The subjects performed exercises in positions corrected by the instructor in terms of maintaining joint alignment, with slightly bent knees and body weight transferred to the forefoot and mid-foot, which served to reduce the propagation of vibrations toward the head.

The Fitvibe Excel Pro vibration platform (Gymna Uniphy, Bilzen, Belgium) used in the research generates vertical vibrations with a frequency in the range of 20–60 Hz and an amplitude of 2 and 4 mm. The amplitude of vibrations (2 mm) and the duration of exercises and rest intervals between them did not change throughout the entire duration of the training. The criterion for changing the training intensity was the increase in the vibration frequency of the vibrating platform (1–12 training (1–4 weeks) 40 Hz; 13–24 training (5–8 weeks) 45 Hz; 25–36 training (9–12 weeks) 50 Hz). In the final part of the training unit, in low positions on the mattress, 3 exercises were performed, 6 repetitions each: in the supine position, extension in the lumbar spine with simultaneous extension in the elbow joints, in the supine position with the lower limbs and buttocks adjacent directly to the wall, the movement of alternating shifting of the heels on the wall, and changing the position from lying on your back with your knees drawn to your chest to sitting (rolling like a ball exercise).

### 2.4. Body Composition Analysis

Prior to the commencement of the main research, body height (BH) was measured using an anthropometer and body composition was estimated with the TANITA BC 418 MA (Tanita, Tokyo, Japan) body composition analyzer to exclude women without normative body weight from the project. During the study, the following morphological components were determined: body mass (BM), body fat percentage (PF), fat mass (FM), fat-free body mass (FFM), and BMI.

### 2.5. Analysis of Nutrition and Assessment of the Level of Physical Activity

Due to the criteria for inclusion in the study, the women determined the amount and type of food intake for 4 working days and 1 weekend in a food diary. Based on the collected data, the average level of food intake was calculated in terms of energy value and the content of proteins, fats, and carbohydrates. Calculations were performed using the computer program Dieta 5 (Institute of Food and Nutrition, Warsaw, Poland) [25]. The level of physical activity was verified using the shortened version of the International Physical Activity Questionnaire (IPAQ) [23].

### 2.6. Blood Collection and Laboratory Indices

Blood was collected in the morning (7.00–9.00) from the basilic vein in a sitting position by a qualified laboratory diagnostician in accordance with the applicable standards. Subjects were fasting. The studies were planned so that blood sampling took place in the follicular phase of the menstrual cycle. In whole blood collected in K_2_EDTA tubes (2 mL) using the Sysmex XN 9000 apparatus (Sysmex, Kobe, Japan), the following indicators were determined: red blood cell count (RBC, 10^6^/µL), hematocrit (Hct, %), hemoglobin (Hb, g/dL), mean erythrocyte hemoglobin mass (MCH, pg), mean erythrocyte hemoglobin concentration (MCHC, g/dL), mean erythrocyte volume (MCV, fl), red blood cell volume distribution (RDW-SD, fl), reticulocyte count (RETC, 10^6^/mm^3^), and platelet count (PLT, 10^3^/µL).

In the blood (4 mL) within 4 h of collection, the rheological properties of the blood were determined using a laser-assisted optical rotational cell analyzer (RR, Mechatronics, Hoorn, The Netherlands) according to the Hardeman method [28]. Erythrocyte deformability was expressed by the elongation index (EI). The following aggregation indices were calculated: aggregation index (AI, in %), aggregation amplitude (AMP, in au), and half time of complete aggregation (T½ in s).

Changes in plasma volume (%∆PV) were estimated using the formula of Dill and Costill [29], modified by Harisson et al. [30]. Calculations were made based on Hb and Hct results using the formula:

%∆PV = 100{(Hb_1_/Hb_2_) · [100 − (Hct_2_ · 0.874)]/[100 − (Hct_1_ · 0.874)] − 1},

where Hb_1_ and Hct_1_ are the baseline values of hemoglobin concentration and hematocrit number, respectively, and Hb_2_ and Hct_2_ are the values of these indicators after exercise.

The concentration of fibrinogen [g/L] was determined in plasma obtained from blood collected in a sodium citrate tube (1.8 mL) by coagulometry using the Siemens BCS XP apparatus (Siemens Healthineers, Marburg, Germany).

### 2.7. Statistical Analysis

SPSS Statistics 24 (IBM) software was used for statistical analysis. The type of distribution was tested with the Kolmogorow–Smirnov test. Differences in anthropometric characteristics and nutrition between the groups at baseline were verified by one-way ANOVA or the Kruskal–Wallis test with corresponding post hoc Sidak or Dunn tests. The student’s *t*-test was used to analyze the differences between the change in plasma volume and group membership. To verify the occurrence of differences between the analyzed biochemical indices and the time of their measurement, and between the groups, a two-factor univariate mixed model ANOVA was performed. In the case of revealing the significance of the time or group effect, multiple (post hoc) comparisons were made using the Sidak test and the NIR. Thanks to the ANOVA test, short-term effects of exercise (comparison of values before and after the first and last training, and values after the first and last training) in EG and CG and long-term effects (comparison of values at rest on the first and last day of the study) in EG, CG, and CON were compared. In each analysis, the results were considered significant at *p* < 0.05.

## 3. Results

### 3.1. Subjects’ Characteristics

As a result of the conducted analyses, statistically significant intergroup differences in body weight and fat-free mass were found. Women from EG were characterized by lower BM and FFM compared to the subjects from the CON group (*p* < 0.05) (Table 2).

### 3.2. Short-Term Effects of Exercise

#### 3.2.1. Hematological Indices

Hb analysis showed a group effect (*p* = 0.009). In EG both before (*p* = 0.001) and after the last training (*p* = 0.031), Hb values were significantly lower than in CG. For MCV, the presence of an interaction effect was found: time × group (*p* < 0.001). In EG, after the last WBVT, there was a significant decrease in MCV compared to the measurement after the first training (*p* = 0.006). MCH changed significantly over time (*p* < 0.001); however, post hoc tests showed no changes for the analyzed measurements. The MCHC also showed a group effect (*p* = 0.002). After the first training session in women with EG, its value was significantly lower than in CG (*p* < 0.001). There was also a group effect for RETC (*p* = 0.027). After the last training in women with EG, the number of RETC was significantly lower compared to CG (*p* = 0.042) (Table 3).

#### 3.2.2. Elongation Index (EI)

EI analysis at a shear stress of 0.3 Pa showed the occurrence of the time effect (*p* < 0.001) and the time x group interaction (*p* < 0.001). In EG, after the last WBVT, the EI value compared to the state after the first training increased significantly (*p* < 0.001). For EI determined at a shear rate of 0.58 Pa, two effects are observed: time (*p* = 0.004) and interaction-time × group (*p* < 0.001). In EG, EI was significantly higher after the last WBVT compared to the state after the first training (*p* < 0.001). A time effect was observed for EI at a shear stress of 1.13 Pa (*p* = 0.03). However, the results of post hoc tests showed no differences for the analyzed measurements (Table 4).

#### 3.2.3. Aggregation Indices and Fibrinogen Concentration

The AMP analysis indicates the presence of a group effect (*p* < 0.001) and an interaction-time × group (*p* = 0.003). For EG, AMP values in measurements I and II (*p* < 0.001), III (*p* = 0.007), and IV (*p* = 0.017) were significantly lower than in CG. In EG, after the last WBVT, there was a significantly higher increase in AMP compared to the state after the first training (*p* = 0.006). There are no significant changes in fibrinogen values (Table 5).

### 3.3. The Effects of a 12-Week Training Program

#### 3.3.1. Hematological Indices

The analysis of the results of hematological tests after 12 weeks of training showed differences for Hb (*p* < 0.001). EG in measurement performed in rest on the last day of training program obtained significantly lower Hb vs. CG (*p* = 0.003). The time effect was demonstrated for MCV (*p* = 0.015). Post hoc tests showed a decrease in MCV in response to repeated WBVT in EG (*p* = 0.015). MCH values changed significantly over time (*p* = 0.006). The post hoc test indicated that these differences apply to EG, where MCH decreased significantly under the influence of repeated WBVT (*p* = 0.006). The time factor also differentiated the MCHC results (*p* = 0.015). A significant increase in MCHC was observed after a series of EG training sessions (*p* = 0.005) (Table 6).

#### 3.3.2. Elongation Index (EI)

The EI analysis showed the occurrence of the time effect for the successively tested shear rates (0.3 Pa *p* < 0.001; 0.58 Pa *p* = 0.048; 1.13 Pa *p* = 0.004). The results of post hoc tests in EG showed a significant increase in EI under the influence of a series of vibration trainings at a shear stress of (0.3 Pa *p* < 0.001; 0.58 Pa *p* < 0.001; 1.13 Pa *p* < 0.001), respectively (Table 7).

#### 3.3.3. Aggregation Indices and Fibrinogen Concentration

Effects for time (*p* = 0.003) and group (*p* < 0.001) for AMP have been demonstrated. A significant increase in AMP under the influence of repeated vibration training was confirmed only in EG (*p* = 0.003). Both in the first (*p* < 0.001) and third (*p* < 0.05) measurements, significant intergroup differences were found, consisting in the fact that EG obtained significantly lower AMP values than CG, while CG obtained significantly higher values than CON. There are no significant changes in fibrinogen values (Table 8).

#### 3.3.4. Changes in the Blood Plasma Volume (ΔPV)

In EG, after the first training, there was a greater decrease in plasma volume than in CG, but these results were not statistically significant (*p* > 0.05). In response to the last training, plasma volume decreased in women with EG, while it increased in women with CG. The results of the conducted analyses showed statistically significant differences between the change in plasma volume during the last training and the examined groups of women (*p* = 0.003). As a result of a series of trainings, plasma volume increased in EG and decreased in CG; however, no significant differences were found in the change in plasma volume after repeated training and group affiliation (*p* > 0.05). (Table 9).

## 4. Discussion

The mechanical action of vibrations generated by vibrating platforms in connection with physical exercises in the context of blood rheology has not been clarified so far. The results of our research show that the effect of repeated and cumulative vibration and physical exercise can be increased blood flow in the vessels. On the other hand, changes in the erythrocyte image after 3-month WBVT testify to the activation of the body’s adaptive mechanisms, presumably as a result of an increase in plasma volume. The greatest advantage of the research presented in this article is that we were the first to address the topic of the influence of WBVT on the rheological properties of blood in young, healthy women. Many authors of studies agree on the improvement of blood flow in the vessels under the influence of WBVT [9,31,32]. One of the discussed factors affecting the kinetics of blood flow is the increase in muscle contraction caused by the tonic vibration reflex [32]. Another possible explanation for this phenomenon is the reduction in blood viscosity under the influence of vibrations and the associated increase in blood flow velocity [33]. In addition, WBVT, through mechanical vibrations, affects the functions of the vessels, reducing the tone and stiffness of the arteries [34]. The increase in blood flow under the influence of WBVT may be related to the effect of pulsatile stress on vascular endothelium, which results in an increase in the activity of endothelial nitric oxide synthase (eNOS) and the concentration of nitric oxide (NO) [8,35].

A well-known and described in the literature phenomenon occurring under the influence of a single physical effort, both submaximal and maximal, is hemoconcentration resulting from at least five different mechanisms: redistribution of erythrocytes in the vascular bed; an increase in the number of red blood cells because of shrinkage of the spleen; enrichment of plasma with proteins of lymphatic origin; loss of water with sweat in the process of thermoregulation; and drainage of water to muscle cells [22,36]. Ahmadizad and El-Sayed, in their study of young, healthy men, noted a parallel decrease in plasma volume and an increase in plasma viscosity, RBC, Hb, and Ht in response to a single session of resistance exercise [37]. The above temporary changes in hemorheological indices confirm the occurrence of the previously described phenomenon of hemoconcentration. Similar results were also obtained in a study by Ramagnoli et al., where the impact of a single aerobic exercise on a cycloergometer was assessed in young, untrained people [15]. In our own research, both the first and last physical training on the vibration platform caused a greater loss of plasma volume percentage compared to the group of women performing physical exercises without the vibration factor, but significant changes were observed only after the last WBVT session. These results may indicate a greater degree of dehydration because of the sweating process in the group of women subjected to vibration training. In addition, in the measurement made before the last training, a significantly lower hemoglobin concentration was observed in women undergoing WBVT, which is probably related to the observed increase in plasma volume because of adaptation to exercise, during which water is lost to maintain a constant body temperature.

The results of studies by Theodorou et al. conducted on a group of healthy middle-aged women did not show any changes in white and red blood cell indices and in the level of platelets, both under the influence of a single exposure to WBV, as well as 2-month vibration training performed three times a week on the axle platform. Exercise duration (6–8 min) and frequency (20–25 Hz) increased gradually, while the vibration amplitude (6 mm) was the same throughout the training [38]. Similarly, in own studies, no significant changes in hematological parameters, including reticulocytes and platelets, were found under the influence of a single WBVT session, which suggests that the applied vibration stimulus was probably insufficient to stimulate the circulatory system and increase the hematopoietic activity of the bone marrow. The adaptive reaction of the body in response to repeated physical exercise on subsequent days is the so-called chronic autohemodilution resulting in a baseline decrease in plasma viscosity and hematocrit level [20,36]. In our own study, in relation to hematological indices, only a slight decrease in the average volume of red blood cells and the average mass of hemoglobin in an erythrocyte was observed after 12 weeks of WBVT. In the case of the MCV index, it is found to decrease after the last training in relation to the state after the first exercise session in combination with WBV. The above effect may indicate long-term changes in the form of water movement in the body between the cell and extracellular fluids, which is confirmed by the increase in %ΔPV.

The level of training may be one of the factors that determines the elasticity of red blood cells [18,39]. Connes et al. showed that the accumulation of lactate in the body, resulting from local hypoxia during exercise above the anaerobic threshold, causes a decrease in physically inactive people, and an increase in erythrocyte deformability in people who train [39]. In this study, single training in both EG and CG did not change the elongation index. Since the effect of WBVT can be considered as the effect of the cumulative effect of vibrations and physical exercises, our own results suggest that in physically inactive people, the inclusion of vibrations in training does not reduce the deformability of red blood cells.

Another phenomenon that has a significant impact on blood circulation is aggregation. Red blood cells at low tangential stress collide with each other, forming clusters called rolls. Erythrocyte aggregates are formed primarily in small blood vessels, where blood flow velocity decreases [20]. It has been shown that a single session of strength training with moderate and submaximal intensity, both at the beginning and at the end of a 6-week training program, increases erythrocyte aggregation [18]. In the studies presented in this article, there were no significant changes in AI and t1/2 after the first and last training session in both study groups, which proves that the applied training protocol in combination with whole-body vibration is a safe form of exercise, as it does not lead to pathophysiological changes in the rheological properties of blood. The obtained intergroup differences in the aggregation amplitude index may result from different levels of body hydration. This factor has been proven to impact blood rheology [40,41]. A valuable supplement to the research would probably be to determine the initial average water content in the body.

Comparing the body’s reaction after the last training in relation to the condition after the first training, a significant increase in the aggregation amplitude index was observed in the study group, possibly as a result of increased water loss during WBVT. It should be emphasized that the analyzed aggregation values were characterized by a large dispersion around the mean, which may indicate a high inter-individual differentiation.

In presented research, in the group of women subjected to repeated WBVT, a slight increase in erythrocyte deformability was observed at low shear stress, which is a positive phenomenon, indicating an improvement in blood flow through the capillaries and an increase in tissue oxygenation in the long-term aspect. The confirmation of the above changes is also the significant increase in EI values observed in these studies at the shear stress of 0.3 and 0.58 Pa in response to the last WBV compared to the state after the first training. Perhaps longer exposure to vibrations would result in a stronger response from the circulatory system. The results of studies by Kilic-Toprak et al. evaluating the effect of resistance training on erythrocyte deformability in young, healthy people showed an increase in EI in the third and fourth week of exercise in relation to the initial values. In addition, in response to the last training conducted in week 12, this parameter increased again [42]. Analyzing our own results, in the group of women exercising on a vibrating platform, in response to repeated exposure to WBVT, a significant increase in the amplitude of aggregation was observed, which can be explained by the processes of slowing down aggregation after training and body dehydration. It should also be emphasized that the other aggregation indices are normal, which means that changes in AMP are small.

The increase in erythrocyte aggregation during strenuous exercise is believed to be related to the increase in plasma fibrinogen levels associated with hemoconcentration [19]. Contrary to our results, the studies of Boyle et al. showed that in young, healthy men, a single 15-min vibrotraining with a frequency of 30 Hz and an amplitude of vibrations of 1.5 mm, combined with squats, increased fibrinolytic activity to a greater extent than the use of exercises alone [43]. In this study, a single physical effort on a vibrating platform did not cause changes in fibrinogen concentration. Since the level of this protein is an independent cardiovascular risk factor, WBVT may be a safer form of exercise for people who are ill or in the high-risk group. It is known that regular physical activity is one of the factors that reduces the concentration of fibrinogen in the blood [44]. As research by Kabaty-Piżuch et al. shows, vibrotherapy can also affect changes in the level of this protein in the blood. The use of 30 treatments using both general and local vibrations in men aged 60–70 resulted in a decrease in the parameters of body fat and the concentration of fibrinogen in the blood. Slight changes in the form of reductions were observed for Hb, RBC, and MCH. However, no changes in erythrocyte aggregation indices were found [45]. Ghazalian, in his studies, did not show significant changes in fibrinogen concentration under the influence of 6-week WBVT with both low and high amplitude and vibration frequency in the range of 25–50 Hz increasing progressively by 5 Hz per week in healthy, young, and physically inactive men patients [46]. In our study, 12-week training on a vibration platform with a low, constant amplitude of 2 mm and an increasing frequency in the range of 40–50 Hz also did not affect changes in fibrinogen concentration. It is also worth noting that both our own study and the study conducted by Ghazalian included healthy people whose index values were within the normal range.

### Study Limitation

The key limitations of the conducted studies include small sample size, significant differences in the size of the study and comparison groups, baseline differences between groups, the lack of randomization, volunteer sampling, and the non-simultaneous inclusion of participants in the study. Another limitation of the research that may affect the obtained results is that the required sample size was not calculated at the project planning stage.

## 5. Conclusions

In young, healthy women after applying a series of 36 trainings in a standing position on a vibrating platform, the process of losing body fluids with sweat increases, blood flow in the vessels improves, and in the long term, the process of supplying oxygen and nutrients to working muscles and other body tissues is improved.

Including WBV in physical training does not increase erythrocyte aggregation and does not negatively affect the level of fibrinogen, which plays a significant role in the pathogenesis of cardiovascular diseases and confirms the safety of this form of physical exercise.

In the described studies, important information could be provided by performing additional determinations of the examined hemorheological and biochemical indicators within 30 min after the end of physical effort. The reasons discussed earlier may also indicate that the load was not sufficiently high during the entire training cycle in both study groups. Therefore, there is a need for further research to understand the mechanism of internal changes in the human body under the influence of WBVT.

## Figures and Tables

**Table 1:** Inclusion and exclusion criteria.

Inclusion Criteria	Exclusion Criteria
Age 18–25 years Female Written consent to participate in the study Low (insufficient) level of physical activity determined based on the International Physical Activity Questionnaire—IPAQ [23] Normal body mass index (BMI) in the range of 18.5–24.9 [24] A diet that does not differ from the recommendations of the Food and Nutrition Institute [25] No contradictions to WBVT confirmed by medical qualifications [26,27]	Smoking tobacco Use of hormonal contraception Diagnosed polycystic ovary syndrome or anovulatory cycles Special and elimination diets up to 3 months before joining the research project Regular intake of antioxidant vitamins and other dietary supplements at least one month before the start of the study

**Table 2:** Characteristics of the subjects.

Parameter	EG (*n* = 17)	CG (*n* = 12)	CON (*n* = 17)	*p*
Age [years]	21.65 ± 1.8	20.17 ± 1.75	19.53 ± 0.72	0.064
BH [cm]	162.76 ± 7.51	164.67 ± 5.94	167.24 ± 4.56	0.187
BM [kg]	56.57 ± 7.18	59.43 ± 6.04	63.29 ± 8.71 ^	0.029
BMI [kg/cm^2^]	21.31 ± 1.87	22.02 ± 2.91	22.57 ± 2.44	0.351
PBF [%]	23.04 ± 6.11	25.62 ± 4.14	26.25 ± 5.76	0.351
FM [kg]	13.34 ± 4.54	15.4 ± 3.85	17.03 ± 5.89	0.119
FFM [kg]	43.24 ± 3.81	44.03 ± 2.84	46.25 ± 3.24 ^	0.013

Values are mean ± standard deviation; body height (BH); body mass (BM); body mass index (BMI); percentage body fat (PBF); fat mass (FM); fat free mass (FFM); ^ significant difference (*p* < 0.05) between the EG/CON.

**Table 3:** Changes in hematological indicators after a single bout of exercise in experimental (EG) and comparison group (CG).

Parameter		I	II	Δ II-I	III	IV	Δ IV-III	Δ IV-II	ANOVA *p* Values (η2)		
									Time	Group	T × G
RBC [10^6^/µL]	EG	4.34 ± 0.3	4.41 ± 0.29	0.07 ± 0.17	4.31 ± 0.27	4.38 ± 0.27	0.07 ± 0.08	−0.03 ± 0.25	0.400 (0.035)	0.130 (0.083)	0.467 (0.031)
	CG	4.49 ± 0.31	4.52 ± 0.34	0.03 ± 0.11	4.53 ± 0.29	4.53 ± 0.29	0 ± 0.09	0.01 ± 0.18			
Hb [g/dL]	EG	12.71 ± 0.68	12.99 ± 0.76	0.28 ± 0.46	12.44 ± 0.66 #	12.7 ± 0.7 #	0.26 ± 0.25	−0.29 ± 0.49	0.132 (0.065)	0.009 (0.227)	0.176 (0.059)
	CG	13.34 ± 0.74	13.41 ± 0.78	0.07 ± 0.31	13.39 ± 0.79	13.34 ± 0.79	−0.05 ± 0.25	−0.07 ± 0.72			
Ht [%]	EG	38 ± 1.93	38.68 ± 2.06	0.68 ± 1.52	37.19 ± 1.97	37.81 ± 1.76	0.62 ± 0.79	−0.87 ± 2.16	0.416 (0.030)	0.098 (0.098)	0.066 (0.102)
	CG	38.73 ± 2.18	38.86 ± 2.51	0.13 ± 1.05	39.33 ± 1.94	39.16 ± 1.91	−0.17 ± 0.85	0.30 ± 2.02			
MCV [fl]	EG	87.75 ± 3.87	87.86 ± 3.53	0.12 ± 0.93	86.46 ± 3.38	86.46 ± 3.4 †	0 ± 0.49	−1.41 ± 1.39	0.111 (0.083)	0.596 (0.011)	<0.001 (0.240)
	CG	86.46 ± 2.82	86.09 ± 2.77	−0.37 ± 0.99	86.89 ± 2.89	86.51 ± 3.04	−0.38 ± 0.68	0.42 ± 1.7			
MCH [pg]	EG	29.35 ± 1.19	29.49 ± 1.14	0.15 ± 0.35	28.92 ± 1.28	29.02 ± 1.29	0.10 ± 0.31	−0.47 ± 0.62	<0.001 (0.202)	0.330 (0.035)	0.233 (0.052)
	CG	29.78 ± 1.07	29.73 ± 1.01	−0.05 ± 0.29	29.59 ± 1.44	29.48 ± 1.44	−0.11 ± 0.62	−0.24 ± 0.82			
MCHC [g/dL]	EG	33.44 ± 0.64	33.58 ± 0.44 #	0.14 ± 0.50	33.46 ± 0.79	33.58 ± 0.75	0.12 ± 0.47	0 ± 0.82	0.107 (0.078)	0.002 (0.298)	0.110 (0.077)
	CG	34.47 ± 0.88	34.53 ± 0.62	0.06 ± 0.72	34.05 ± 0.85	34.06 ± 0.78	0.01 ± 0.31	−0.47 ± 0.55			
RDW-SD [fl]	EG	41.1 ± 3.16	40.96 ± 2.94	−0.14 ± 0.94	40.6 ± 2.28	40.6 ± 2.28	−0.01 ± 0.40	−0.37 ± 2.5	0.743 (0.015)	0.165 (0.070)	0.127 (0.068)
	CG	39.12 ± 2.49	38.88 ± 2.23	−0.23 ± 0.23	40.17 ± 3.73	40.17 ± 3.73	−0.33 ± 0.63	0.95 ± 2.43			
PLT [10^3^/µL]	EG	263.82 ± 55.6	286.47 ± 62.54	22.65 ± 17.51	275.71 ± 61.04	285.94 ± 54.49	10.24 ± 12.57	−0.53 ± 48.63	0.126 (0.068)	0.152 (0.075)	0.723 (0.016)
	CG	301.42 ± 61.7	307.58 ± 65.8	6.17 ± 19.45	310.58 ± 63.56	315.25 ± 65.05	4.67 ± 14.07	7.67 ± 57.07			
RETC [10^6^/mm^3^]	EG	0.05 ± 0.01	0.05 ± 0.01	0 ± 0.01	0.05 ± 0.01	0.05 ± 0.01 #	0 ± 0	0 ± 0.01	0.110 (0.084)	0.027 (0.169)	0.678 (0.011)
	CG	0.06 ± 0.02	0.06 ± 0.02	0 ± 0.01	0.06 ± 0.01	0.06 ± 0.01	0 ± 0.01	0 ± 0.01			

Values are mean ± standard deviation; η2 = eta squared; G × T: group-by-time interaction; red blood cell count (RBC); hemoglobin (Hb); hematocrit (Ht); mean erythrocyte volume (MCV); mean erythrocyte hemoglobin mass (MCH); mean erythrocyte hemoglobin concentration (MCHC); red blood cell volume distribution (RDW-SD); platelet count (PLT); reticulocyte count (RETC); I—measurement performed before the first exercise training; II—measurement performed after the first exercise training; III—measurement performed before the last exercise training; IV—measurement performed after the last exercise training; # significant difference (*p* < 0.05) between the EG/CG; † significant difference (*p* < 0.05) from measurement II.

**Table 4:** Changes in elongation index (EI) at various levels of shear stress after a single bout of exercise in experimental (EG) and comparison group (CG).

Shear Stress [Pa]		I	II	Δ II-I	III	IV	Δ IV-III	Δ IV-II	ANOVA *p* Values (η2)		
									Time	Group	T × G
(0.3)	EG	0.002 ± 0.011	0.001 ± 0.011	−0.001 ± 0.006	0.022 ± 0.019	0.025 ± 0.017 †	0.003 ± 0.023	0.024 ± 0.014	<0.001 (0.341)	0.062 (0.093)	<0.001 (0.283)
	CG	0.036 ± 0.01	0.036 ± 0.01	0 ± 0.006	0.039 ± 0.008	0.036 ± 0.008	−0.003 ± 0.005	0 ± 0.008			
(0.58)	EG	0.049 ± 0.011	0.048 ± 0.009	−0.001 ± 0.005	0.06 ± 0.008	0.06 ± 0.01 †	0 ± 0.009	0.012 ± 0.009	0.004 (0.168)	0.096 (0.098)	<0.001 (0.328)
	CG	0.068 ± 0.006	0.069 ± 0.007	0.001 ± 0.005	0.067 ± 0.006	0.066 ± 0.01	−0.001 ± 0.006	−0.004 ± 0.009			
(1.13)	EG	0.118 ± 0.011	0.119 ± 0.011	0.001 ± 0.004	0.126 ± 0.009	0.125 ± 0.009	−0.001 ± 0.008	0.006 ± 0.012	0.03 (0.091)	0.624 (0.002)	0.158 (0.069)
	CG	0.137 ± 0.008	0.139 ± 0.008	0.001 ± 0.005	0.138 ± 0.008	0.139 ± 0.012	0 ± 0.006	0 ± 0.117			
(2.19)	EG	0.221 ± 0.014	0.223 ± 0.015	0.001 ± 0.005	0.224 ± 0.019	0.221 ± 0.019	−0.003 ± 0.019	−0.001 ± 0.023	0.942 (0.005)	0.532 (0.009)	0.963 (0.002)
	CG	0.24 ± 0.009	0.241 ± 0.01	0.001 ± 0.006	0.24 ± 0.008	0.24 ± 0.01	−0.001 ± 0.008	−0.001 ± 0.012			
(4.24)	EG	0.33 ± 0.016	0.331 ± 0.016	0.001 ± 0.005	0.326 ± 0.025	0.322 ± 0.028	−0.004 ± 0.030	−0.009 ± 0.031	0.645 (0.016)	0.754 (0.005)	0.598 (0.019)
	CG	0.345 ± 0.01	0.345 ± 0.011	0.001 ± 0.007	0.345 ± 0.008	0.346 ± 0.007	0.001 ± 0.008	0 ± 0.012			
(8.24)	EG	0.412 ± 0.018	0.413 ± 0.017	0.001 ± 0.008	0.409 ± 0.025	0.404 ± 0.031	−0.004 ± 0.029	−0.009 ± 0.032	0.876 (0.008)	0.486 (0.025)	0.478 (0.027)
	CG	0.428 ± 0.01	0.429 ± 0.011	0.001 ± 0.008	0.429 ± 0.008	0.431 ± 0.007	0.001 ± 0.008	0.002 ± 0.012			
(15.98)	EG	0.482 ± 0.017	0.483 ± 0.016	0.001 ± 0.007	0.478 ± 0.023	0.473 ± 0.029	−0.004 ± 0.026	−0.009 ± 0.028	0.923 (0.006)	0.398 (0.046)	0.267 (0.048)
	CG	0.491 ± 0.011	0.491 ± 0.012	−0.001 ± 0.009	0.495 ± 0.01	0.495 ± 0.008	0.001 ± 0.009	0.004 ± 0.014			
(31.03)	EG	0.535 ± 0.014	0.536 ± 0.013	0 ± 0.006	0.531 ± 0.02	0.527 ± 0.025	−0.004 ± 0.024	−0.008 ± 0.023	0.792 (0.009)	0.303 (0.039)	0.256 (0.048)
	CG	0.536 ± 0.012	0.536 ± 0.014	0 ± 0.009	0.538 ± 0.011	0.539 ± 0.009	0.002 ± 0.011	0.003 ± 0.016			
(60.3)	EG	0.569 ± 0.015	0.569 ± 0.013	0 ± 0.005	0.567 ± 0.017	0.563 ± 0.021	−0.004 ± 0.020	−0.006 ± 0.021	0.235 (0.053)	0.368 (0.030)	0.084 (0.101)
	CG	0.563 ± 0.017	0.564 ± 0.021	0.001 ± 0.014	0.572 ± 0.011	0.596 ± 0.081	0.023 ± 0.074	0.032 ± 0.079			

Values are mean *±* standard deviation; η2 = eta squared; G × T: group-by-time interaction; I—measurement performed before the first exercise training; II—measurement performed after the first exercise training; III—measurement performed before the last exercise training; IV—measurement performed after the last exercise training; † significant difference (*p* < 0.001) from measurement II.

**Table 5:** Changes in aggregation indices and fibrinogen concentration after a single bout of exercise in experimental (EG) and comparison group (CG).

Parameter		I	II	Δ II-I	III	IV	Δ IV-III	Δ IV-II	ANOVA *p* Values (η2)		
									Time	Group	T × G
AMP [au]	EG	13.08 ± 1.59 ##	12.89 ± 1.96 ##	−0.19 ± 2.29	15.17 ± 2.14 #	15.06 ± 2.33 # †	−0.12 ± 2.24	2.17 ± 3.34	0.104 (0.076)	<0.001 (0.698)	0.003 (0.162)
	CG	17.44 ± 1.91	18.24 ± 1.91	0.80 ± 2.63	17.63 ± 2.27	17.08 ± 1.65	−0.55 ± 1.28	−1.16 ± 1.45			
T½ [s]	EG	2.36 ± 0.77	2.17 ± 0.69	−0.19 ± 0.47	2.51 ± 0.74	2.57 ± 1.45	0.06 ± 0.88	0.40 ± 1.34	0.387 (0.038)	0.392 (0.028)	0.736 (0.009)
	CG	2.54 ± 0.52	2.57 ± 0.56	0.03 ± 0.33	2.64 ± 0.97	2.75 ± 0.95	0.12 ± 0.37	0.19 ± 0.98			
AI [%]	EG	62.02 ± 7.45	63.59 ± 7.07	1.57 ± 5.00	60.45 ± 6.28	61.1 ± 9.38	0.65 ± 5.05	−2.49 ± 8.43	0.530 (0.028)	0.392 (0.028)	0.567 (0.019)
	CG	59.74 ± 4.17	59.52 ± 4.32	−0.22 ± 2.53	59.67 ± 7.66	58.4 ± 7.46	−1.27 ± 2.78	−1.13 ± 7.47			
fibrinogen [g/L]	EG	2.57 ± 0.42	2.77 ± 0.54	0.20 ± 0.24	2.6 ± 0.43	2.7 ± 0.45	0.10 ± 0.15	−0.07 ± 0.46	0.113 (0.071)	0.865 (0.001)	0.351 (0.032)
	CG	2.68 ± 0.58	2.74 ± 0.62	0.05 ± 0.20	2.53 ± 0.4	2.58 ± 0.41	0.05 ± 0.13	−0.16 ± 0.541			

Values are mean ± standard deviation; η2 = eta squared; G × T: group-by-time interaction; aggregation amplitude (AMP); half time of complete aggregation (T½); aggregation index (AI); I—measurement performed before the first exercise training; II—measurement performed after the first exercise training; III—measurement performed before the last exercise training; IV—measurement performed after the last exercise training; # significant difference (*p* < 0.05) between the EG/CG; ## significant difference (*p* < 0.001) between the EG/CG; † significant difference (*p* < 0.05) from measurement I.

**Table 6:** Changes in hematological indicators after 12-week training program in experimental (EG) and comparison group (CG) and at an interval of 3 months in control group (CON).

Parameter		I	III	Δ III-I	ANOVA *p* Values (η2)		
					Time	Group	T × G
RBC [10^6^/µL]	EG	4.34 ± 0.3	4.31 ± 0.27	−0.03 ± 0.27	0.681 (0.004)	0.126 (0.092)	0.593 (0.024)
	CG	4.49 ± 0.31	4.53 ± 0.29	0.05 ± 0.1			
	CON	4.46 ± 0.24	4.48 ± 0.29	0.03 ± 0.21			
Hb [g/dL]	EG	12.71 ± 0.68	12.44 ± 0.66 #	−0.27 ± 0.78	0.356 (0.020)	<0.001 (0.550)	0.400 (0.042)
	CG	13.34 ± 0.74	13.39 ± 0.79	0.05 ± 0.5			
	CON	12.85 ± 0.67	12.8 ± 0.71	−0.05 ± 0.6			
Ht [%]	EG	38 ± 1.93	37.19 ± 1.97	−0.81 ± 2.35	0.385 (0.018)	0.064 (0.120)	0.144 (0.086)
	CG	38.73 ± 2.18	39.33 ± 1.94	0.6 ± 1.22			
	CON	38.75 ± 1.39	38.2 ± 1.89	−0.55 ± 1.85			
MCV [fl]	EG	87.75 ± 3.87	86.46 ± 3.38 *	−1.29 ± 1.57	0.015 (0.131)	0.759 (0.013)	0.534 (0.014)
	CG	86.46 ± 2.82	86.89 ± 2.89	0.43 ± 1.57			
	CON	87.06 ± 3.94	85.36 ± 4.32	−1.7 ± 3.05			
MCH [pg]	EG	29.35 ± 1.19	28.92 ± 1.28 *	−0.42 ± 0.38	0.006 (0.160)	0.245 (0.063)	0.613 (0.023)
	CG	29.78 ± 1.07	29.59 ± 1.44	−0.18 ± 0.76			
	CON	28.88 ± 1.64	28.62 ± 1.98	−0.26 ± 0.83			
MCHC [g/dL]	EG	33.44 ± 0.64	33.46 ± 0.79 *	0.02 ± 0.38	0.015 (0.178)	0.134 (0.073)	0.895 (0.000)
	CG	34.47 ± 0.88	34.05 ± 0.85	−0.42 ± 0.76			
	CON	33.15 ± 0.92	33.51 ± 0.91	0.36 ± 0.83			
RDW-SD [fl]	EG	41.1 ± 3.16	40.6 ± 2.28	−0.5 ± 2.85	0.794 (0.002)	0.366 (0.046)	0.146 (0.086)
	CG	39.12 ± 2.49	40.17 ± 3.73	1.05 ± 3.05			
	CON	41.15 ± 2.34	40.29 ± 2.32	−0.86 ± 1.99			
PLT [10^3^/µL]	EG	263.82 ± 55.6	275.71 ± 61.04	11.88 ± 42.51	0.110 (0.058)	0.255 (0.062)	0.966 (0.002)
	CG	301.42 ± 61.7	310.58 ± 63.56	9.17 ± 52.35			
	CON	268.06 ± 67.45	281.94 ± 76.63	13.88 ± 49.45			
RETC [10^6^/mm^3^]	EG	0.05 ± 0.01	0.05 ± 0.01	0 ± 0.01	0.309 (0.024)	0.184 (0.076)	0.122 (0.093)
	CG	0.06 ± 0.02	0.06 ± 0.01	−0.01 ± 0.01			
	CON	0.05 ± 0.02	0.06 ± 0.02	0 ± 0.01			

Values are mean *±* standard deviation; η2 = eta squared; G × T: group-by-time interaction; red blood cell count (RBC); hemoglobin (Hb); hematocrit (Ht); mean erythrocyte volume (MCV); mean erythrocyte hemoglobin mass (MCH); mean erythrocyte hemoglobin concentration (MCHC); red blood cell volume distribution (RDW-SD); platelet count (PLT); reticulocyte count (RETC); I—measurement performed in rest on the first day of training program; III—measurement performed in rest on the last day of training program; # significant difference (*p* < 0.05) between the EG/CG; * significant difference (*p* < 0.05) from measurement I.

**Table 7:** Changes in elongation index (EI) at various levels of shear stress after 12-week training program in experimental (EG) and comparison group (CG) and at an interval of 3 months in control group (CON).

Shear Stress [Pa]		I	III	Δ III-I	ANOVA *p* Values (η2)		
					Time	Group	T × G
(0.3)	EG	0 ± 0.01	0.02 ± 0.02 *	0.020 ± 0.020	<0.001 (0.505)	0.267 (0.003)	0.456 (0.057)
	CG	0.04 ± 0.01	0.04 ± 0.01	0.003 ± 0.010			
	CON	0.02 ± 0.01	0.04 ± 0.02	0.024 ± 0.001			
(0.58)	EG	0.049 ± 0.011	0.06 ± 0.008 *	0.011 ± 0.010	0.048 (0.088)	0.498 (0.002)	0.132 (0.045)
	CG	0.068 ± 0.006	0.067 ± 0.006	−0.001 ± 0.008			
	CON	0.062 ± 0.008	0.06 ± 0.008	−0.002 ± 0.008			
(1.13)	EG	0.118 ± 0.01	0.126 ± 0.009 *	0.008 ± 0.011	0.004 (0.300)	0.092 (0.094)	0.187 (0.031)
	CG	0.137 ± 0.008	0.138 ± 0.008	0.001 ± 0.010			
	CON	0.129 ± 0.009	0.118 ± 0.009	−0.011 ± 0.012			
(2.19)	EG	0.221 ± 0.014	0.224 ± 0.019	0.002 ± 0.021	0.734 (0.002)	0.692 (0.057)	0.298 (0.049)
	CG	0.24 ± 0.009	0.24 ± 0.008	0.001 ± 0.010			
	CON	0.233 ± 0.012	0.201 ± 0.011	−0.033 ± 0.015			
(4.24)	EG	0.33 ± 0.016	0.326 ± 0.025	−0.004 ± 0.078	0.085 (0.056)	0.154 (0.042)	0.365 (0.008)
	CG	0.345 ± 0.01	0.345 ± 0.00	0 ± 0.010			
	CON	0.341 ± 0.014	0.291 ± 0.014	−0.050 ± 0.016			
(8.24)	EG	0.412 ± 0.018	0.409 ± 0.025	−0.003 ± 0.029	0.876 (0.008)	0.429 (0.035)	0.478 (0.027)
	CG	0.428 ± 0.01	0.429 ± 0.008	0.002 ± 0.008			
	CON	0.424 ± 0.016	0.372 ± 0.015	−0.052 ± 0.016			
(15.98)	EG	0.482 ± 0.017	0.478 ± 0.023	−0.004 ± 0.026	0.258 (0.007)	0.387 (0.043)	0.621 (0.001)
	CG	0.491 ± 0.011	0.495 ± 0.01	0.003 ± 0.008			
	CON	0.491 ± 0.016	0.445 ± 0.015	−0.046 ± 0.016			
(31.03)	EG	0.535 ± 0.014	0.531 ± 0.02	−0.004 ± 0.022	0.148 (0.062)	0.095 (0.123)	0.082 (0.156)
	CG	0.536 ± 0.012	0.538 ± 0.011	0.002 ± 0.008			
	CON	0.542 ± 0.012	0.503 ± 0.012	−0.039 ± 0.012			
(60.3)	EG	0.569 ± 0.015	0.567 ± 0.017	−0.002 ± 0.021	0.087 (0.078)	0.081 (0.110)	0.166 (0.094)
	CG	0.563 ± 0.017	0.572 ± 0.011	0.009 ± 0.011			
	CON	0.576 ± 0.012	0.543 ± 0.014	−0.033 ± 0.012			

Values are mean *±* standard deviation; η2 = eta squared; G × T: group-by-time interaction; I—measurement performed in rest on the first day of training program; III—measurement performed in rest on the last day of training program; * significant difference (*p* < 0.001) from measurement I.

**Table 8:** Changes in aggregation indices and fibrinogen concentration after 12-week training program in experimental (EG) and comparison group (CG) and at an interval of 3 months in control group (CON).

Parameter		I	III	Δ III-I	ANOVA *p* Values (η2)		
					Time	Group	T × G
AMP [au]	EG	13.08 ± 1.59 ##	15.17 ± 2.14 * #	2.09 ± 2.94	0.003 (0.205)	<0.001 (0.550)	0.170 (0.087)
	CG	17.44 ± 1.91 &&	17.63 ± 2.27 &	0.2 ± 2.65			
	CON	13 ± 2.13	14.59 ± 1.99	1.59 ± 2.18			
T½ [s]	EG	2.36 ± 0.77	2.51 ± 0.74	0.15 ± 0.92	0.597 (0.007)	0.802 (0.011)	0.827 (0.009)
	CG	2.54 ± 0.52	2.64 ± 0.97	0.1 ± 1.04			
	CON	2.44 ± 0.97	2.4 ± 0.97	−0.04 ± 0.65			
AI [%]	EG	62.02 ± 7.45	60.45 ± 6.28	−1.56 ± 7.71	0.769 (0.002)	0.723 (0.016)	0.693 (0.018)
	CG	59.74 ± 4.17	59.67 ± 7.66	−0.07 ± 8.42			
	CON	61.23 ± 7.96	61.87 ± 8.52	0.64 ± 5.69			
fibrynogen [g/L]	EG	2.57 ± 0.42	2.6 ± 0.43	0.04 ± 0.4	0.792 (0.002)	0.988 (0.001)	0.422 (0.039)
	CG	2.68 ± 0.58	2.53 ± 0.4	−0.16 ± 0.56			
	CON	2.55 ± 0.46	2.62 ± 0.61	0.07 ± 0.49			

Values are mean *±* standard deviation; η2 = eta squared; G × T: group-by-time interaction; aggregation amplitude (AMP); half time of complete aggregation (T½); aggregation index (AI); I—measurement performed in rest on the first day of training program; III—measurement performed in rest on the last day of training program; # significant difference (*p* < 0.05) between the EG/CG; ## significant difference (*p* < 0.001) between the EG/CG; & significant difference (*p* < 0.05) between the CG/CON; && significant difference (*p* < 0.001) between the CG/CON; * significant difference (*p* < 0.001) from measurement I.

**Table 9:** Changes in blood plasma volume [%ΔPV] after a single bout of exercise and 12-week training program in experimental group (EG) and comparison group (CG).

			*p*
%ΔPV1	EG	−2.85 ± 5.36	0.215
	CG	−0.62 ± 3.40	
%ΔPV2	EG	−2.77 ± 2.66	0.003
	CG	0.64 ± 2.92	
%ΔPV3	EG	3.65 ± 9.42	0.137
	CG	−1.01 ± 5.54	

Values are mean *±* standard deviation; %ΔPV1—change after first exercise bout; %ΔPV2—change after last exercise bout; %ΔPV3—change after 12-week training program.

## Data Availability

The data presented in this study are available on request from the corresponding authors.
